# Design and Calibration of Microarrays as Universal Transcriptomic Environmental Biosensors

**DOI:** 10.1002/cfg.466

**Published:** 2005-04

**Authors:** J. S. Almeida, D. J. McKillen, Y. A. Chen, P. S. Gross, R. W. Chapman, G. Warr

**Affiliations:** 1 Department of Biostatistics Bioinformatics, and Epidemiology, Medical University of South Carolina 135 Cannon Street Suite 303, P.O. Box 250835 Charleston SC 29425 USA; 2 Department of Biochemistry and Molecular Biology Medical University of South Carolina Charleston SC USA; 3 Marine Biomedicine and Environmental Science Center Medical University of South Carolina Charleston SC USA; 4 South Carolina Department of Natural Resources Charleston SC USA


**Comparative and Functional Genomics**
*Comp Funct Genom* 2005; **6**: 132–137.
Published online in Wiley InterScience (www.interscience.wiley.com). **DOI:** 10.1002/cfg.466

**Conference Paper**

### Design and calibration of microarrays as universal transcriptomic environmental biosensors

J. S. Almeida[1]*, D. J. McKillen[2], Y. A. Chen[1], P. S. Gross[2,3], R. W. Chapman[3,4] and G. Warr[2,3]

[1]*Department of Biostatistics, Bioinformatics, and Epidemiology, Medical University of South Carolina, Charleston, SC, USA*
[2]*Department of Biochemistry and Molecular Biology, Medical University of South Carolina, Charleston, SC, USA*
[3]*Marine Biomedicine and Environmental Science Center, Medical University of South Carolina, Charleston, SC, USA*
[4]*South Carolina Department of Natural Resources, Charleston, SC, USA*

**Correspondence to:*
*J. S. Almeida, Department of Biostatistics, Bioinformatics, and Epidemiology, Medical University of South Carolina, 135 Cannon Street, Suite 303, P.O. Box 250835, Charleston, SC 29425, USA.*
*E-mail: almeidaj@musc.edu*

Received: 23 January 2005
Accepted: 7 February 2005

**Keywords:** **transcriptomics; microarrays; biosensors; bioinformatics**

### Introduction

The use of sentinel species, by associating physiological responses or population dynamics to external parameters, has a long tradition in environmental studies (LeBlanc and Bain, 1997; Zelikoff, 1998; Cajaraville *et al.*, 2000; Komar, 2001; Golden and Rattner, 2003; Blanco and Cooper, 2004; Moore *et al.*, 2004). In its simplest configuration this may correspond to looking for the presence of biological indicators of environmental quality (LeBlanc and Bain, 1997) but it can also be conFigured for specific toxic contaminants (Cajaraville *et al.*, 2000) or infectious agents (Komar, 2001). Moreover, after realizing that single gene diseases are the exception rather than the rule, the biomedical field is engaged in a gold rush to find transcriptomic and proteomic markers for the diagnosis and prognosis of systemic diseases such as cancer and autoimmune diseases (Chanin *et al.*, 2004; Devauchelle and Chiocchia, 2004; Kuo *et al.*, 2004; Li *et al.*, 2004; Khalil and Hill, 2005). Putting the two together and attempting to use molecular profiles as a sensitive indicator for the status of sentinel species comes as the logical next step (Figure 1). However, a number of serious methodological hurdles remain in the way of realizing what might otherwise be a straightforward proposition. Foremost is the unresolved functional interpretation of the transcriptomic signal itself, particularly when oligonucleotide microarray technologies are used, as different platforms produce 'jaw droppingly' (Marshall, 2004) little concordance (Tan *et al.*, 2003). cDNA microarrays appear to fare better with regard to precision, albeit they have a reputation of low reproducibility and are even less concordant with oligonucleotide microarray results (Woo *et al.*, 2004). Finally, the calibration of transcriptomic biosensors requires recourse to advanced, computationally intensive, pattern recognition algorithms and the collection of sufficiently representative calibration data (Rhodes and Chinnaiyan, 2004). Both premises are not

Copyright © 2005 John Wiley & Sons, Ltd.

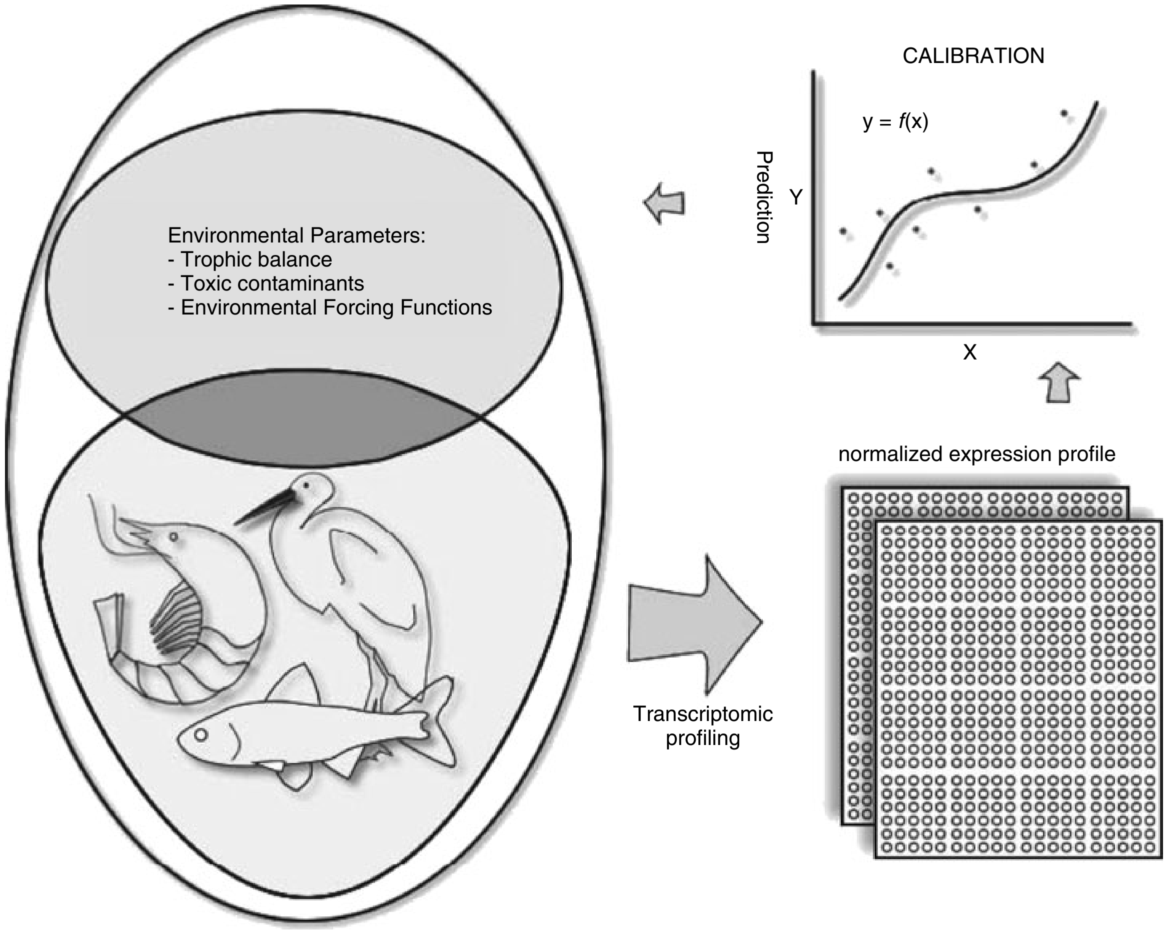

**Figure 1.** Universal environmental transcriptomic biosensor concept: expression microarrays probe the transcriptome of selected sentinel species for calibration, by machine learning, to the target environmental parameters trivial, as illustrated by the missteps of the intensive search for biomarkers for clinical applications (Baggerly *et al*., 2004), an endeavour of much smaller complexity and with access to ample resources.

### Sentinel species

Although there is a long tradition of using sentinel species in environmental science, it does not always follow that the traditional sentinel species are a good choice for transducing environmental forcing functions as recognizable changes in their physiology. In particular the use of keystone species (Brown *et al*., 2001) is not necessarily effective, and the choice should instead focus on species that are physically in contact with media shared by most of the community. This makes aquatic or amphibian species natural choices (Gracey and Cossins, 2003), particularly when they feed off primary producers, e.g. by filtering them out of the large volumes of water (Tanguy *et al*., 2002; Manduzio *et al*., 2004) or by having a life cycle that puts them in contact with a diversity of niches. Similarly, different tissues will have different sensitivity to the target environmental parameter, e.g. the immune system is typically the organ of choice to detect the presence of toxic contaminants (Zelikoff, 1998).

The use of prokaryotes is for the most part excluded from consideration, given the labile nature of their messenger RNA and the difficulty in sampling material from individual species (Dharmadi and Gonzalez, 2004). But for this, prokaryotes and microbial communities in general

Copyright © 2005 John Wiley & Sons, Ltd.

might be a promising choice. Examples abound in the literature where proteomic (Wolf *et al.*, 2003) or lipidomic (Almeida and Noble, 2000; Batten and Scow, 2003; Peacock *et al.*, 2004) profiles from microbial communities were reliably used as biosensors. This assessment may soon change, as indicated by the extension of molecular biology methods usually applied to single organisms or homogeneous cultures to entire biological communities (Venter *et al.*, 2004).

### Transducing the transcriptomic signal

Ideally, the transcriptomic signal would be both reproducible and interpretable. The technology has matured to the point where methodological reproducibility, but not concordance, is achieved by both cDNA and oligonucleotide microarray technologies (Tan *et al.*, 2003). Interpretation is a much harder challenge, as even the most basic understanding of which gene is being targeted by a known probe is not certain: the same report describes how different oligonucleotide microarray platforms will generate signals for the same sample with very little concordance. This problem will not prevent the identification of a transcriptomic marker signal, but it will make more difficult the investigation of its biological basis. In this context, microarrays are not the only available transcriptomic profiling technique and developments in multiplexing PCR-based approaches may be a promising, and cost-effective, alternative (Tian *et al.*, 2004).

With regard to array technology, the use of long coding DNA strands instead of short oligomers presently holds more potential for probing environmental signals for two reasons. First, in spite of the fact that the number of sequenced genomes is fast increasing, it still includes very few of the most promising sentinel aquatic organisms, particularly as regards invertebrates. Second, cDNA microarrays target the transcriptome directly, since they are manufactured by spotting amplified transcripts, instead of relying on short oligomers designed to be collectively specific for the expression of a gene, which is likely to produce multiple splicing variants. Therefore, a widely used procedure to probe the transcriptome for a physiological response to the environment (including infection) is to isolate and sequence expressed sequence tags (ESTs) by

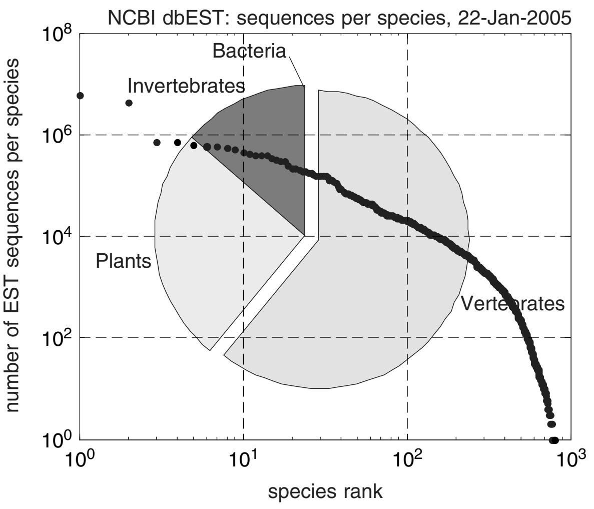

**Figure 2.** Distribution of expressed sequence tag (EST) sequences in the dbEST repository of the National Center for Biotechnology Information between the major taxonomic groups (pie chart in the background). The plot in the foreground describes the distribution of numbers of EST sequences per species. These plots were produced by downloading the summary files directly from dbEST at: **http://www.ncbi.nlm.nih.gov/dbEST**

subtractive hybridization (Snell *et al.*, 2003; Munir *et al.*, 2004). The pursuit of EST projects is now commonplace, as reflected by the 367 species for which there are more than 1000 ESTs described in GenBank's dbEST (Figure 2).

### Biosensor design and manufacture

The selection of sequences to use as probes relies on well established procedures that seek to maximize specificity (hybridization to the desired target sequence) while minimizing the sensitivity (cross-hybridization to other unrelated reverse transcribed RNA sequences). The methodology is fairly well established (for a comprehensive description, see Wit and McClure, 2004; Stekel, 2003) and will not be expanded here beyond recalling that it relies on a sequence analysis procedure to identify sequences that are unique and are not prone to autohybridization. The extent of probe selection, e.g. the number of probes spotted in the microarray, has also been the object of study where a general agreement between the sequence composition, contiguity and functional annotation for probe selection from a EST database could be

Copyright © 2005 John Wiley & Sons, Ltd. 

defined (Chen *et al.*, 2004). There is, however, one consideration worth discussing further — the hybridization models themselves. In fact, the computational tools used to pursue this selection rely on a set of over-simplistic approaches to predict hybridization. Non-specific hybridization (or cross-hybridization) is known to occur in both oligonucleotide and cDNA platforms. Several studies were conducted to model expression intensities, based on binding kinetics using the physical properties or oligo composition in Affymetrix oligonucleotide microarrays (Hekstra *et al.*, 2003; Held *et al.*, 2003; Zhang *et al.*, 2003) and concluded that univariate models fall short of explaining the complexity apparent in the results. Non-specific binding (cross-hybridization) is an even more complex problem for cDNA microarrays because of the length of the probes (Kothapalli *et al.*, 2002). The probe sequences spotted on the arrays are frequently the ESTs collected by subtractive hybridization, which are often not fully sequenced. Several univariate studies were performed to correlate the hybridization intensities and sequence characteristics between the probe–target pair for cDNA microarrays (Evertsz *et al.*, 2001; Xu *et al.*, 2001; Miller *et al.*, 2002), these studies reached the same (and expected) conclusion that sequences sharing a high percentage identity have a higher chance to cross-hybridize with each other. However, all these models contain numerous exceptions that cannot be accommodated by the univariate analyses. To the author's best knowledge, no systematic multivariate predictive model exists for cDNA microarray hybridization experiments.

In conclusion, the design of microarrays currently relies too narrowly on uniparametric models of the sequence. The recent reporting of appallingly little concordance between microarray platforms (Tan *et al.*, 2003) has raised the awareness that there remain major gaps in the understanding of the hybridization process and the manufacturing procedure that need to be better understood. The overview above focuses on microarray technology but it is noteworthy that other transcription profiling techniques exist. Again, a good starting point to consider alternatives is the biomarker identification for multigenic diseases such as cancer (Ahmed, 2002).

### Calibration of the transcriptomic response

Independently of the transcriptomic profiling method chosen and the ability to correctly identify the transcripts targeted, inferring environmental properties from the transcriptome of one organism will depend on a profile (multi-parametric) rather than on the expression of a single gene. Furthermore, the complexity of processes, biotic and abiotic, involved will cause that dependency to be highly non-linear. This scenario is familiar for the identification of proteomic and transcriptomic clinical biomarkers as well as in the use of lipidomic microbial biomarkers for environmental parameters. This combination of multiparametric, complex, non-linear properties converts the calibration of the transcriptomic response into an exercise of pattern recognition.

Pattern recognition for the calibration of transcriptomic biosensors has the particular characteristic that the limiting condition will likely be the relatively small number of parameters when compared with the number of transcripts probed, particularly if microarrays are being used. Furthermore, the use of dimensionality reduction techniques would be detrimental for the calibration, as those procedures target the representation of the variability in the signal, not the variability that is associated with the target environmental parameters. Consequently, some form of variable selection, e.g. selection of a smaller subset of transcriptomic signals, is necessary. In contrast to the situation with microarrays, when a technique is used that probes a number of transcripts under the 100 mark (for most desktop systems under 30 candidates is a more realistic scenario), an exhaustive search of the best combination of parameters is feasible, as we have recently illustrated for RT-PCR biomarker selection (Mitas *et al.*, 2005). However, the number of possibilities would be unreasonable for a similar approach to microarray results. In that case some type of variable selection procedure is needed. Given the interdependency between parameters, the variables selected are likely to be reported as being an unstable set (Li *et al.*, 2004), an observation that is apparent even when the much simpler multilogistic regression is applied to mostly non-molecular parameters (Austin and Tu, 2004).

Several approaches exist that would enable non-linear pattern recognition, which is also often

Copyright © 2005 John Wiley & Sons, Ltd. 

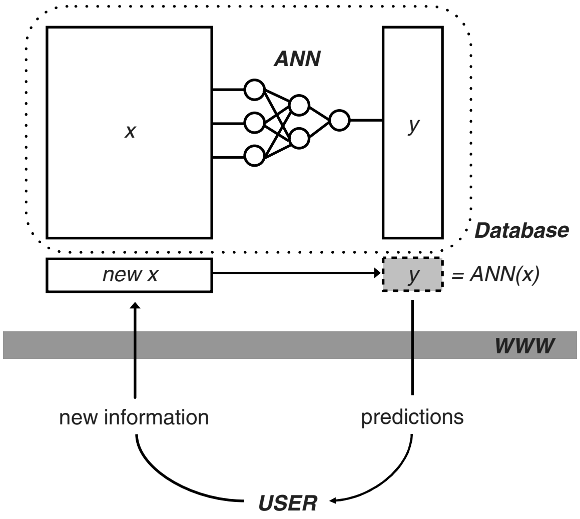

**Figure 3.** Calibration of the microarray environmental biosensor is a continuous, web-based procedure. It relies on a database of microarray profiles (*x*) and environmental parameters (*y*) that is used to maintain an updated machine learning (artificial neural network, ANN, in this schema) representation of the association between the two. New microarray results can be submitted for prediction of the target parameters and production of predictions creates an opportunity for further validation, and refinement of the calibration, by subsequent submission of the observed outcome, when and if available described as a machine learning procedure. Among those, artificial neural networks (Almeida, 2002), support vector machines (Man *et al.*, 2004) and Bayesian inference (Ochs *et al.*, 2004; Khalil and Hill, 2005), are particularly popular. Implicit in this approach, the machine learning calibration of microarray environmental biosensors is a continuous procedure that reflects the latest availability of the data, as described in Figure 3. The application of these analytical tools requires a data-management infrastructure geared for both data warehousing and model validation specialized to the target ecosystem. One example is the Marine Genomics consortium, where the investigators are focusing their research efforts to develop transcriptomic microarray biosensors for use in the marine environment (**http://marinegenomics.org**).

### Acknowledgements

This work was partially supported by the National Science Foundation (MCB0315393), the National Marine Fisheries Service (NA03NMF4720362), the South Carolina Sea Grant Consortium (R/MT-6), United States Department of Agriculture (USDA NRICGPCSREES/AREA 2002-35201-11620), National Oceanic and Atmospheric Administration Oceans and Human Health Iniative (Award #85133) and the South Carolina Department of Natural Resources. This is publication #20 of the Marine Biomedicine and Environmental Sciences program at MUSC. Any opinions, findings, and conclusions or recommendations expressed in this material are those of the author(s) and do not necessarily reflect the views of the supporting bodies mentioned herein.

### References

Ahmed FE. 2002. Molecular techniques for studying gene expression in carcinogenesis. *J Environ Sci Health C Environ Carcinog Ecotoxicol Rev* **20**(2): 77–116.

Almeida JS. 2002. Predictive non-linear modeling of complex data by artificial neural networks. *Curr Opin Biotechnol* **13**(1): 72–76.

Almeida JS, Noble PA. 2000. Neural computing in microbiology. *J Microbiol Methods* **43**(1): 1–2.

Austin PC, Tu JV. 2004. Automated variable selection methods for logistic regression produced unstable models for predicting acute myocardial infarction mortality. *J Clin Epidemiol* **57**(11): 1138–1146.

Baggerly KA, Morris JS, Coombes KR. 2004. Reproducibility of SELDI-TOF protein patterns in serum: comparing datasets from different experiments. *Bioinformatics* **20**(5): 777–785.

Batten KM, Scow KM. 2003. Sediment microbial community composition and methylmercury pollution at four mercury mine-impacted sites. *Microb Ecol* **46**(4): 429–441.

Blanco GA, Cooper EL. 2004. Immune systems, geographic information systems (GIS), environment and health impacts. *J Toxicol Environ Health B Crit Rev* **7**(6): 465–480.

Brown JH, Whitham TG, Morgan Ernest SK, Gehring CA. 2001. Complex species interactions and the dynamics of ecological systems: long-term experiments. *Science* **293**(5530): 643–650.

Cajaraville MP, Bebianno MJ, Blasco J, *et al.* 2000. The use of biomarkers to assess the impact of pollution in coastal environments of the Iberian Peninsula: a practical approach. *Sci Total Environ* **247**(2–3): 295–311.

Chanin TD, Merrick DT, Franklin WA, Hirsch FR. 2004. Recent developments in biomarkers for the early detection of lung cancer: perspectives based on publications 2003 to present. *Curr Opin Pulmon Med* **10**(4): 242–247.

Chen YA, McKillen DJ, Wu S, *et al.* 2004. Optimal cDNA microarray design using expressed sequence tags for organisms with limited genomic information. *BMC Bioinformat* **5**(1): 191.

Devauchelle V, Chiocchia G. 2004. [What place for DNA microarray in inflammatory diseases?]. *Rev Med Interne* **25**(10): 732–739.

Dharmadi Y, Gonzalez R. 2004. DNA microarrays: experimental issues, data analysis, and application to bacterial systems. *Biotechnol Prog* **20**(5): 1309–1324.

Evertsz EM, Au-Young J, Ruvolo MV, *et al.* 2001. Hybridization cross-reactivity within homologous gene families on glass cDNA microarrays. *Biotechniques* **31**(5): 1182, 1184, 1186 passim.

Golden NH, Rattner BA. 2003. Ranking terrestrial vertebrate species for utility in biomonitoring and vulnerability to

Copyright © 2005 John Wiley & Sons, Ltd. 

environmental contaminants. *Rev Environ Contam Toxicol* **176**: 67–136.

Gracey AY, Cossins AR. 2003. Application of microarray technology in environmental and comparative physiology. *Annu Rev Physiol* **65**: 231–259.

Hekstra D, Taussig AR, Magnasco M, Naef F. 2003. Absolute mRNA concentrations from sequence-specific calibration of oligonucleotide arrays. *Nucleic Acids Res* **31**(7): 1962–1968.

Held GA, Grinstein G, Tu Y. 2003. Modeling of DNA microarray data by using physical properties of hybridization. *Proc Natl Acad Sci USA* **100**(13): 7575–7580.

Khalil IG, Hill C. 2005. Systems biology for cancer. *Curr Opin Oncol* **17**(1): 44–48.

Komar N. 2001. West Nile virus surveillance using sentinel birds. *Ann NY Acad Sci* **951**: 58–73.

Kothapalli R, Yoder SJ, Mane S, Loughran TP, Jr. 2002. Microarray results: how accurate are they? *BMC Bioinformat* **3**(1): 22.

Kuo WP, Whipple ME, Epstein JB, *et al*. 2004. Deciphering gene expression profiles generated from DNA microarrays and their applications in oral medicine. *Oral Surg Oral Med Oral Pathol Oral Radiol Endod* **97**(5): 584–591.

LeBlanc GA, Bain LJ. 1997. Chronic toxicity of environmental contaminants: sentinels and biomarkers. *Environ Health Perspect* **105**(suppl 1): 65–80.

Li L, Tang H, Gong J, *et al*. 2004. Data mining techniques for cancer detection using serum proteomic profiling. *Artif Intell Med* **32**(2): 71–83.

Man MZ, Dyson G, Johnson K, Liao B. 2004. Evaluating methods for classifying expression data. *J Biopharm Stat* **14**(4): 1065–1084.

Manduzio H, Monsinjon T, Galap C, *et al*. 2004. Seasonal variations in antioxidant defences in blue mussels (*Mytilus edulis*) collected from a polluted area: major contributions in gills of an inducible isoform of Cu/Zn-superoxide dismutase and of glutathione S-transferase. *Aquat Toxicol* **70**(1): 83–93.

Marshall E. 2004. Getting the noise out of gene arrays. *Science* **306**(5696): 630–631.

Miller NA, Gong Q, Bryan R, *et al*. 2002. Cross-hybridization of closely related genes on high-density macroarrays. *Biotechniques* **32**(3): 620–625.

Mitas M, Almeida JS, Mikhitarian K, *et al*. 2005. Accurate discrimination of Barrett's esophagus and esophageal adenocarcinoma using a quantitative three-tiered algorithm and multimarker real-time RT-PCR. *Clin Cancer Res* (in press).

Moore MN, Depledge MH, Readman JW, Paul Leonard DR. 2004. An integrated biomarker-based strategy for ecotoxicological evaluation of risk in environmental management. *Mutat Res* **552**(1–2): 247–268.

Munir S, Singh S, Kaur K, Kapur V. 2004. Suppression subtractive hybridization coupled with microarray analysis to examine differential expression of genes in virus infected cells. *Biol Proced Online* **6**: 94–104 (http://www.biologicalprocedures.com).

Ochs MF, Moloshok TD, Bidaut G, Toby G. 2004. Bayesian decomposition: analyzing microarray data within a biological context. *Ann NY Acad Sci* **1020**: 212–226.

Peacock AD, Chang YJ, Istok JD, *et al*. 2004. Utilization of microbial biofilms as monitors of bioremediation. *Microb Ecol* **47**(3): 284–292.

Rhodes DR, Chinnaiyan AM. 2004. Bioinformatics strategies for translating genome-wide expression analyses into clinically useful cancer markers. *Ann N Y Acad Sci* **1020**: 32–40.

Snell TW, Brogdon SE, Morgan MB. 2003. Gene expression profiling in ecotoxicology. *Ecotoxicology* **12**(6): 475–483.

Stekel D. 2003. Microarray Bioinformatics. Cambridge University Press, Cambridge.

Tan PK, Downey TJ, Spitznagel EL, *et al*. 2003. Evaluation of gene expression measurements from commercial microarray platforms. *Nucleic Acids Res* **31**(19): 5676–5684.

Tanguy A, Boutet I, Bonhomme F, *et al*. 2002. Polymorphism of metallothionein genes in the Pacific oyster *Crassostrea gigas* as a biomarker of response to metal exposure. *Biomarkers* **7**(6): 439–450.

Tian H, Cao L, Williams S, *et al*. 2004. Multiplex mRNA assay using electrophoretic tags for high-throughput gene expression analysis. *Nucleic Acids Res* **32**(16): e126.

Venter JC, Remington K, Heidelberg JF, *et al*. 2004. Environmental genome shotgun sequencing of the Sargasso Sea. *Science* **304**(5667): 66–74.

Wit E, McClure J. 2004. *Statisticals for Microarrays: Design, Analysis and Inference*. John Wiley & Sons Ltd, Chichester.

Wolf G, Almeida JS, Crespo JG, Reis MA, *et al*. 2003 Monitoring of biofilm reactors using natural fluorescence fingerprints. *Water Sci Technol* **47**(5): 161–167.

Woo Y, Affourtit J, Daigle S, *et al*. 2004. A comparison of cDNA, oligonucleotide, and Affymetrix GeneChip gene expression microarray platforms. *J Biomol Tech* **15**(4): 276–284.

Xu W, Bak S, Decker A, *et al*. 2001. Microarray-based analysis of gene expression in very large gene families: the cytochrome P450 gene superfamily of *Arabidopsis thaliana*. *Gene* **272**(1–2): 61–74.

Zelikoff JT. 1998. Biomarkers of immunotoxicity in fish and other non-mammalian sentinel species: predictive value for mammals? *Toxicology* **129**(1): 63–71.

Zhang L, Miles MF, Aldape KD. 2003. A model of molecular interactions on short oligonucleotide microarrays. *Nature Biotechnol* **21**(7): 818–821.

Copyright © 2005 John Wiley & Sons, Ltd. 
